# Management of Gynaecological Chronic Pelvic Pain in the Emergency Setting— A Scoping Review

**DOI:** 10.1111/ajo.70132

**Published:** 2026-04-20

**Authors:** Lucinda Peacock, Therlma Nyamutora, Katie Palmer, Tim Andrews, Bill Lord, Kelly‐Ann Bowles

**Affiliations:** ^1^ New South Wales Ambulance Sydney New South Wales Australia; ^2^ Monash University Clayton Victoria Australia; ^3^ Peninsula Health Frankston Victoria Australia; ^4^ Ambulance Victoria Doncaster Victoria Australia

**Keywords:** chronic pain, paramedicine, pelvic pain, research, scoping review

## Abstract

**Background:**

Gynaecological chronic pelvic pain (CPP) has a high prevalence among adult women. This pain can be debilitating and life‐impacting, affecting quality of life across all dimensions of health. Many barriers to specialised care exist, leading women to rely on unscheduled general care like emergency departments or in the out‐of‐hospital.

**Objective:**

The review aimed to investigate evidence regarding pain management for women presenting to acute, non‐specialist settings for gynaecological CPP. Secondary aims were to identify areas of suboptimal care and potential for improvement of patient outcomes.

**Methods:**

A scoping review was conducted using the Joanna Briggs Institute scoping review framework with the PRISMA extension. Academic and grey literature were searched.

**Results:**

Initially, 1563 records were identified, with 50 from grey literature and 801 from citation screening. Nineteen were included for descriptive analysis. Key themes were pharmacological agents, holistic care and ongoing care. Appraisal of the literature showed varying quality.

**Conclusion:**

This scoping review highlights the need to define clinicians' roles in managing gynaecological CPP exacerbations in acute, non‐specialist settings. It identifies gaps in best‐practice pain assessment, management and clinician education, with guidelines and recommendations often of poor quality. Effective CPP management requires a multidisciplinary and biopsychosocial approach and, despite limitations, clinicians can enhance knowledge and practice scope to improve patient outcomes.

## 1 Introduction

### 1.1 Background

Chronic pelvic pain (CPP), also called persistent pelvic pain, is an umbrella term covering many complex conditions related to or perceived in structures in the pelvic region [1]. The pain classified as lasting over 3–6 months affects approximately 15%–20% of women [1, 2, 3]. This term includes specific conditions or affected organ groups including bladder pain syndrome/urological pelvic pain, fibromyalgia and myofascial syndromes/neuromusculoskeletal pelvic pain and irritable bowel syndrome (IBS)/gastrointestinal pelvic pain [2, 4]. In addition to pathophysiology in the pelvic region, CPP may be categorised as nociplastic in nature in line with the International Association for the Study of Pain's definition, which refers to pain as an ‘unpleasant sensory and emotional experience associated with, or resembling that associated with actual or potential tissue damage’ [5]. Furthermore, CPP may also involve central sensitization, a process where persistent peripheral pain (e.g., hyperalgesia or allodynia) contributes to the chronicity of symptoms [1, 6]. Women with CPP endure frequent episodes of severe, life‐impacting pain disrupting activities of daily living [7, 8]. The sequelae of CPP extend beyond physical manifestations of pain, potentially affecting all health dimensions including psychological and social wellbeing [9, 10]. Women with CPP often report more anxiety, depression and reduced health‐related quality of life [6]. Moreover, CPP is strongly associated with prior trauma (physical, emotional or sexual), and pain can be sustained through psychosocial factors including kinesiophobia and maladaptive coping strategies [6, 11, 12]. Treatment demands a multidisciplinary and multidimensional approach involving both pharmacological and non‐pharmacological therapies [10, 13].

Gynaecological pelvic pain conditions, the focus of this scoping review, refer to CPP originating from or perceived to impact female reproductive organs [3, 4]. Pain perception in relation to gynaecologic aetiologies may be modulated or informed by central sensitisation as described above [14]. Common conditions associated with gynaecological CPP include dysmenorrhea, endometriosis, adenomyosis, uterine fibroids and polycystic ovarian syndrome [2, 3, 4, 15].

True prevalence of gynaecological CPP is difficult to determine due to varied definitions and categorisations of pain. Many estimates cover CPP overall without separating gynaecologic versus non‐gynaecological disorders or specific conditions themselves (e.g., endometriosis). Regardless, gynaecologic aetiologies have been reported to occur in approximately half of those with CPP [2]. Endometriosis, the leading gynaecologic CPP cause is estimated to affect one in nine Australian women [2, 16].

Significant challenges to accessible care including long diagnostic delays and cost barriers may result in patients seeking care at public, generalised facilities including emergency departments (EDs) [17]. Patients with chronic pain seek care in the acute setting for a multitude of reasons which may include exacerbations of existing pain, feelings of loss of control over pain and an onsets of new pain [18]. Healthcare professionals (HCPs) in these settings, including paramedics, may become involved in care when patients feel other options for symptom management are unavailable [17, 19, 20]. Though these settings may lack resources for comprehensive care due to limitations, including education of clinicians and time constraints, they often serve as a stop‐gap, providing expedient or emergency care before definitive treatment [20].

The aim of this scoping review was to document the existing evidence pertaining to the management of CPP (gynaecological aetiologies) in acute care settings. The research team sought to evaluate the strengths and weaknesses of these settings in providing multi‐dimensional, holistic care to women presenting with pain associated with gynaecological CPP. The primary objective of this review is to document the provision of pain management practices, with a secondary objective of identifying suboptimal care, defined as gaps in addressing key management dimensions and opportunities to enhance care. This study explores current evidence, assesses deficiencies and proposes practical recommendations to guide improved care delivery within the acute care setting for women with gynaecological CPP.

## 2 Methods

### 2.1 Protocol and Registration

The authors originally intended to research how the pain of patients with gynaecological CPP is managed in the out‐of‐hospital setting. However, preliminary searches showed no literature for this population exclusively in that setting. Thus, the context broadened to the ‘acute, non‐specialist setting’ also encompassing clinical settings including the ED. The difficulty in locating paramedic‐specific literature prompted the authors to select a scoping review, due to its capacity in identifying broader themes, concepts and knowledge gaps within an evolving field of research [21, 22].

This review followed the Joanna Briggs Institute (JBI) scoping review framework [22]. The PRISMA extension for scoping reviews (PRISMA‐ScR) checklist is provided in Appendix S1 [23]. The protocol, including the search strategy was developed in June 2024 and registered with the Open Science Framework for transparency in August 2024 [24].

### 2.2 Ethics Statement

Not applicable.

### 2.3 Language

The authors recognise the need for gender‐inclusive language when discussing pathologies typically associated with cisgender women. Language involves complex considerations; binary sexed categorisation can further perpetuate health inequities for marginalised groups like trans and intersex people whilst inaccurate use of gender‐inclusive language may misrepresent statistics and individual experiences [25].

The target population of this review is ‘women with CPP from a gynaecological cause’. The terms ‘woman/women’ and ‘female’ appear throughout, reflective of corresponding literatures reporting. These terms encompass all individuals Assigned Female at Birth (AFAB) experiencing gynaecological CPP regardless of gender identity [25].

This review set no publication year limit for articles—thus, many older articles screened lacked specific definitions of ‘female’ or ‘woman/women’, potentially excluding trans, gender‐diverse and nonbinary individuals. To account for gender‐diverse individuals, the search strategy for population, found in Appendix S2, used condition‐specific terms like ‘endometriosis’ without gender‐qualified phrases such as ‘women’ with endometriosis’.

### 2.4 Eligibility Criteria

#### 2.4.1 PCC

Per the JBI scoping review guide, the Population, Concept, Context (PCC) framework was utilised: Population—AFAB persons with CPP from a gynaecological cause, Concept—how pain is managed or perceived, including patient and provider views and interventions, Context—The acute, non‐specialist setting including the ED and the out‐of‐hospital setting [22]. Search terms stemmed from defining areas of interest within the PCC. These were discussed and refined by all authors, reaching a joint consensus. The PCC then informed the inclusion and exclusion criteria.

#### 2.4.2 Inclusion and Exclusion Criteria

To fulfil the ‘population’ criteria, studies needed reference to gynaecological CPP, defined as pain lasting over a 3–6‐month period involving or perceived to impact female reproductive organs, either generally or within specific conditions (e.g., endometriosis, adenomyosis, dysmenorrhea). Non‐gynaecological CPP (e.g., musculoskeletal, urological) causes were only included if gynaecological aetiology was also noted. In the acute care setting, presentations typically involve acute flares of chronic pain (acute‐on‐chronic or sub‐acute exacerbations) with some screened literature referring to these flares as ‘acute pelvic pain’. Given this ambiguity, literature was included regardless of semantic categorisation, if all other eligibility criteria were met.

Regarding ‘concept’; it is understood that pain may not be a symptom that each individual diagnosed with conditions associated with CPP experiences [26, 27]. Therefore, for the purpose of this review as outlined in the ‘concept’, pain as a symptom has to be explicitly mentioned in each publication. Literature that discussed management of CPP in an emergency setting without any reference to the assessment and treatment of pain was excluded. Additionally, patients who present with gynaecological CPP may suffer from symptoms other than pain (e.g., bladder and bowel dysfunction); however, as pain is the focus of this review, other symptomology will be excluded within the discussion.

The context of this study is the acute, non‐specialist setting, largely encompassing the emergency department and the out‐of‐hospital setting. Papers which specify a more generalised audience were judged on individual merit as to if the therapies are applicable to the acute, non‐specialist setting.

### 2.5 Information Sources

The search strategy was developed, discussed and refined primarily by the principal author (LP) with input from all members of the research team, comprising experienced academics. To systematically and comprehensively identify all relevant documents, a three‐step search strategy was utilised. The following databases were searched: Medline, Emcare, CINAHL and AMED. There was no date range restriction for the search; however, only literature translated into English was eligible for screening. Literature was considered for inclusion regardless of its design. Grey literature was then searched by inputting key areas of interest into the Google search engine and screening the first 50 results (5 pages) as recommended by the JBI framework [22]. Finally, forwards and backwards citation screening was conducted with all included literature.

### 2.6 Search

Databases and grey literature were searched in December 2024, with citation searching taking place subsequently. The full search strategy is available in Appendix S2.

### 2.7 Selection of Sources of Evidence

Following the search, citations were exported into EndNote and then imported into Covidence (an online literature screening tool). Covidence automatically removed duplicates, with remaining duplicates manually removed by two researchers (LP and TN). The review process consisted of two layers of screening; title and abstract and full‐text screening. As recommended by the JBI framework, the initial screening process was pilot tested to ensure consistency between reviewers [22]. This was done by researchers LP and TN screening the same sample of 50 abstracts, discussing the results and amending the screening criteria accordingly before full title and abstract screening took place [22].

Both researchers independently conducted title and abstract screening where screening criteria were applied—conflicts were resolved by the two researchers with oversight by researcher KB. Full‐text screening was then completed by researchers LP and TN, leaving remaining articles for data extraction. Grey literature and citation searches were screened by both reviewers using the same criteria as the primary search.

### 2.8 Data‐Charting Process and Data Items

Data was extracted from included literature by the principal author (LP) and another researcher (TN), using a data extraction template created by the principal author, informed by the JBI framework [22]. Data extracted included author name, year of publication, country of origin, aims, study design, key findings or themes, diagnosis and clinical setting. The extracted data was then exported from Covidence into a table on ‘Google Docs’ for analysis and to allow collaboration between authors.

### 2.9 Critical Appraisal of Individual Sources of Evidence

Articles were appraised as recommended by the JBI approach and the PRISMA‐SCR checklist [22, 23]. Guidelines were assessed independently by three researchers (LP, KB and KP) using the Appraisal of Guidelines for Research and Evaluation (AGREE II) instrument, whilst case studies, expert opinions and qualitative studies were independently assessed by two researchers (LP and KP) using their respective JBI checklist [28, 29]. A suitable appraisal method for literature categorised as ‘clinical reviews’ was not identified.

### 2.10 Synthesis of Results

To summarise the key themes of the included articles, a content analysis was undertaken whereby findings of the articles were categorised according to their management strategies, as informed by the PCC and review question [30]. Authors LP and TN inductively coded the text line‐by‐line according to management principles [30]. Codes were then discussed with other members of the research team; analytical themes were generated and finalised [30].

## 3 Results

### 3.1 Selection of Sources of Evidence

**FIGURE 1:**
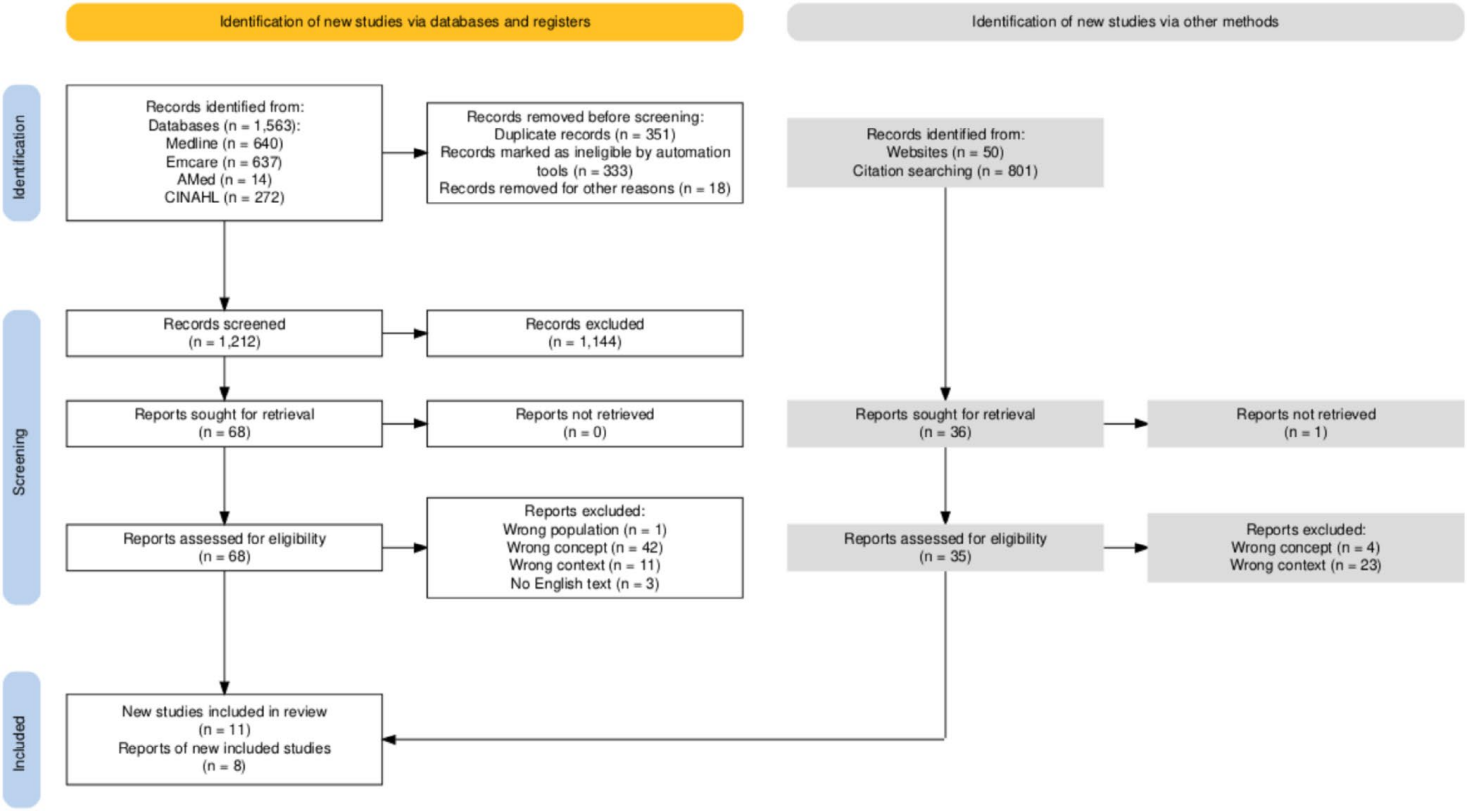
PRISMA flow diagram.

A total of 1563 records were identified through database searching conducted in December of 2024 (Medline: 640, Emcare: 637, AMED: 14, CINAHL: 272). The removal of 351 duplicates left 1212 articles eligible for title and abstract screening. Upon completion of the initial screen, 68 articles met the criteria for inclusion in the full‐text screening phase where 11 articles fulfilled eligibility criteria and were included for analysis in the review. A manual search of Google for grey literature yielded 50 articles for screening, seven of which were included in the review. Forwards and backwards citation searching led to the screening of an additional 801 articles, with one being deemed eligible for inclusion in the review. A total of 19 articles were eligible for analysis. See Figure 1 for PRISMA. Full characteristic description of included studies can be found below in Table 1.

**TABLE 1:** Characteristics of included articles.

Author(s) and year	Country	Methodology	Aims	Key findings/themes	Diagnosis and setting
Baines and Allen [31]	United States of America	Clinical review	Diagnosis, treatment, disposition and referral of endometriosis in the emergency department setting.	Referral—to gynaecologist or surgery consult Analgesia—NSAIDs	Endometriosis Emergency department
Bowler et al. [32]	Australia	Guideline	Acute management of pelvic pain flares in the emergency department.	Analgesia—stepwise and multimodal with avoidance of opioids Psychosocial considerations—stressors, reassurance, acknowledgement of pain Non‐pharmacological management—breathing, mobilisation Referral—primary healthcare provider, gynaecologist, pain clinic	CPP Emergency department
Cirilli and Cipot [33]	United States of America	Clinical review	To familiarise emergency department physicians with the presentation and management of vaginal bleeding and pelvic pain.	Analgesia—NSAIDs as first line Hormonal therapy—initiated in ED following consultation Referral—gynaecologist	Uterine fibroids, dysmenorrhea and endometriosis Emergency department
Dason et al. [34]	Canada	Guideline	Evidence‐based management and diagnosis of adenomyosis.	Hormonal therapy—oral contraceptives as a first‐line then GnRH agonists Analgesics—NSAIDs	Adenomyosis Multiple disciplines including emergency physicians and registered nurses
Faculty of Pain Medicine ANZCA [35]	Australia	Statement paper	To enhance the ability of primary care/frontline clinicians to identify and manage pelvic pain effectively.	Psychosocial considerations—‘whole person’ approach Analgesics—NSAIDs Hormonal therapy—hormonal suppression and oral contraceptives Referral—multidisciplinary pain programs Education—Upskilling of all clinicians who encounter CPP	CPP and endometriosis Aimed at all HCP's who encounter CPP, in media release also refers to those in generalised acute/primary care settings
Forcier [36]	United States of America	Clinical review	Describe urgent gynaecologic causes of pelvic pain in adolescent females presenting to the ED.	Analgesics—NSAIDs Hormonal therapy—oestrogen and/or progestin hormones Referral—gynaecologists and ‘adolescent sensitive clinics’	Dysmenorrhea, CPP and endometriosis Emergency department
Hauswald and Kerr [37]	United States of America	Clinical review	Assessment and management of multiple aetiologies of pelvic pain.	Non‐pharmacological management—position of comfort, gentle transport (prehospital) Analgesics—NSAIDs Hormonal therapy—‘Hormonal manipulation’	Pelvic inflammatory disease, ovarian pain and endometriosis Emergency department and out‐of‐hospital setting
Javadian and Shobeiri [38]	United States of America	Clinical review	Pelvic pain investigation and management principles.	Referral—pain clinic if opioids are required Analgesics—NSAIDs and avoidance of opioids Hormonal therapy—oral contraceptives and hormonal replacement therapy	CPP, endometriosis, adenomyosis and pelvic inflammatory disease Aimed at ‘generalists’ who manage CPP, refers to acute settings
Keeler et al. [39]	United States of America	Clinical review	Education on the pathophysiology, assessment and management of endometriosis for nurses.	Hormonal therapy—in combination with other analgesics Non‐pharmacological analgesia—heating pads, massage, breathing techniques Psychosocial considerations—empathetic discussion, self‐advocacy of patients Analgesia—NSAIDs	Endometriosis Aimed at all nurses who treat women with endometriosis, emphasises nurses who don't work in specialist settings
Nadeau et al. [40]	United States of America	Clinical review	Outline how endometriosis should be investigated and treated in the emergency department.	Analgesics—NSAIDs, ketorolac, trometamol, morphine Hormonal therapy—for which referral is required Referral—general practitioner or gynaecologist	Endometriosis Emergency department
Nadeau et al. [41]	United States of America	Case study	To educate ED clinicians on the variable symptomology of endometriosis to more effectively treat patients.	Analgesics—NSAIDs Hormonal therapy—hormonal contraception or progestin‐only as first‐line treatments	Endometriosis Emergency department
National Institute for Health and Care Excellence [42]	United Kingdom	Guideline	To provide advice on management of endometriosis in primary/frontline healthcare settings.	Analgesics—acetaminophen and NSAIDs as first line. Referral if other analgesics are required. Psychosocial considerations—long‐term support across multiple health domains Referral—specialists including surgery, gynaecologists, fertility and pain management clinics Hormonal therapy—oral contraceptive pill	Endometriosis Aimed at multiple disciplines but makes direct reference to HCP's who see women who ‘first present’ in primary and emergency care settings.
North Metropolitan Health Service [43]	Australia	Guideline	To provide guidance on the management of patients presenting with an acute exacerbation of chronic pelvic pain to the emergency centre.	Psychosocial considerations—trauma‐informed care principles, empathetic responses Analgesics—NSAIDs, acetaminophen, neuropathic medications and avoidance of opioids Referral—gynaecological outpatient, pelvic pain clinic or GP Hormonal therapy—commenced in the acute setting if appropriate	Endometriosis, CPP, dysmenorrhea and ovarian pain Emergency centre
Powell [44]	United States of America	Clinical review	To educate HCP's on the aetiology and management of both gynaecologic and non‐gynaecologic causes of CPP in adolescents who present to primary care.	Non‐pharmacological management—complementary/alternative medicine interventions including acupuncture Psychosocial considerations—affirming and empathic statements, psychosocial counselling Hormonal therapy—oral contraceptives and GnRH agonists	Endometriosis, pelvic inflammatory disease and ovarian cysts Multiple disciplines including emergency settings
Queensland Health [45]	Australia	Guideline	To give providers in the acute setting management principles when treating women and girls with flares of CPP.	Non‐pharmacological management—low stimulus, privacy, heat/cold pack, TENS, breathing Psychosocial considerations—biopsychosocial assessment, trauma‐informed, culturally safe Analgesics—simple analgesia for dysmenorrhea, multimodal (anxiolytics, neuropathics, antidepressants), opioid avoidance Referral—primary provider Education—patient education	CPP Acute care settings
Roman et al. [46]	Sweden	Qualitative study	Describing patient experience and expectations related to emergency visits for women with endometriosis.	Analgesia – advocates for, delays of analgesics cause undue suffering Perception—variation between treating HCP, provider disbelief led to inferior analgesia Psychosocial considerations—Empathetic care from nurses improved experience, patients expressed desire for comprehensive plans across biopsychosocial model Non‐pharmacological management—positioning (lying down), heating pads Education—limited knowledge by HCPs may be associated with decreased care, stating education would improve care received	Endometriosis Emergency department
Royal Australian and New Zealand College of Obstetricians and Gynaecologists [47]	Australia	Guideline	Evidence‐based CPG for diagnosis and management of endometriosis in Australian healthcare settings.	Analgesics—NSAIDs and acetaminophen as first line, avoidance/hesitancy of opioids Hormonal therapy—as analgesic adjuvant Referral—gynaecologist Nonpharmacologic management—understanding why women turn to alternative treatments (self‐advocacy), acupuncture, TENS Psychosocial considerations—behavioural/psychological management including CBT, trauma‐informed care principles	Endometriosis Multiple disciplines, refers to both primary (initial settings used as an entry into healthcare system) and secondary (specialist settings)
Steele [48]	United States of America	Clinical review	To provide guidance on the approach toward opioid management of CPP in the OBGYN and emergency department settings.	Referral—pain management centres, tertiary referral centres Analgesics—opioid avoidance	CPP Both OBGYN and emergency department settings
Stenberg et al. [49]	United States of America	Case study	To present the case of spinal block as analgesia in the emergency department for a patient with endometriosis associated pain.	Analgesics—ultrasound‐guided erector spinae nerve block, opioid hesitance Referral—gynaecologist Non‐pharmacological management—positioning	Endometriosis Emergency department

### 3.2 Characteristics of Sources of Evidence

The publication dates ranged between 1989 and 2024. The majority of the included literature originated from the United States of America (*n* = 11) and Australia (*n* = 5). The prevalent methodology utilised in the included literature was review articles (*n* = 9) followed by guidelines (*n* = 6). Two case studies, a statement paper and a qualitative study were also included. The practice settings varied with 13 articles directed at healthcare professionals working in the emergency department. One article specified two settings: the emergency department setting and the out‐of‐hospital setting, the only to directly reference paramedics. The majority of the articles discussed management of endometriosis. The next most common condition referenced was CPP. Six articles discussed the management of multiple conditions.

#### 3.2.1 Management Practices

**FIGURE 2:**
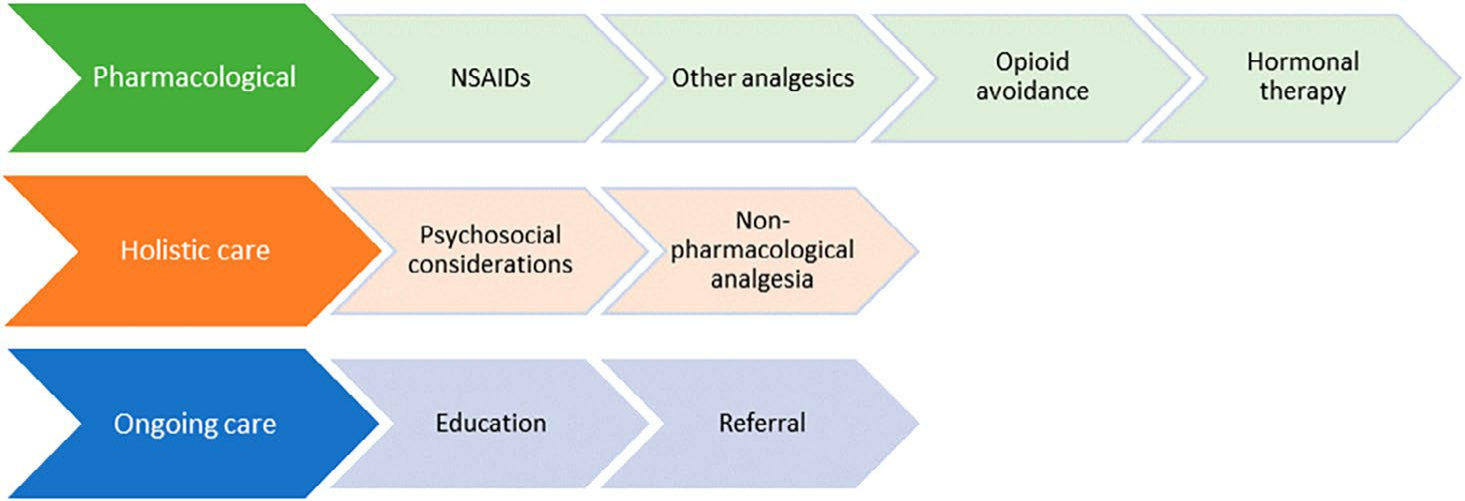
Overview of management practices themes.

Three broad areas of management were identified in this review via content analysis of the codes: (1) pharmacological agents, (2) holistic care and (3) ongoing care. A diagram depicting the association between codes and broader themes is shown in Figure 2. In contrast, suboptimal care was identified when articles failed to address one or more of these key areas.

##### 3.2.1.1 Pharmacological Agents

The use of non‐steroidal anti‐inflammatories (NSAIDs) as first‐line or primary analgesia was the most common pharmacological intervention suggested by 13 articles [32, 33, 34, 35, 36, 37, 38, 39, 40, 41, 42, 43]. Several studies outline the use of NSAIDs without including recommendations of other pharmacological analgesics [31, 33, 34, 35, 36, 37, 39, 41]. Hormonal therapy such as oral contraceptives (oestrogen and/or progestin) and gonadotropin‐releasing hormone (GnRH) agonists were recommended as a standalone therapy or as an adjunct to suppress the menstrual cycle in an effort to provide symptomatic relief by 13 articles [31, 33, 34, 35, 36, 37, 38, 39, 40, 41, 42, 44, 47]. Other analgesics such as spinae nerve block, opioids, acetaminophen, neuropathic medications and anxiolytics were mentioned by eight articles to varying degrees [31, 32, 40, 42, 43, 45, 47, 49]. Seven articles expressed hesitance to or an avoidance of using opioids in the acute, non‐specialist setting [32, 38, 43, 45, 47, 48, 49].

##### 3.2.1.2 Holistic Care

Nine articles suggested that clinicians consider psychological and social factors in their management of pain such as psychiatric care, reassuring and validating the patients' experience of pain, trauma‐informed care and using empathic and affirmative language [32, 35, 39, 42, 43, 44, 45, 46, 47]. Non‐pharmacological analgesics options were presented by nine of the articles, including the use of breathing techniques, positioning, relaxation, gentle mobilisation and transport, heating pads, complementary or alternative medicine, acupuncture and transcutaneous electrical nerve stimulation (TENS) [32, 35, 39, 42, 43, 44, 45, 46, 47].

##### 3.2.1.3 Ongoing Care

Referring patients to other services such as general practitioners (GP's), specialists including obstetrician gynaecologists (OBGYNs) or pain specialists was highlighted by 13 articles [31, 32, 33, 35, 36, 38, 40, 42, 43, 45, 47, 48, 49]. Upskilling and continued education of practitioners were only recommended by two articles [35, 45]. One article mentioned the importance of continued education for patients [45].

##### 3.2.1.4 Suboptimal Care

Eight of the 19 articles, including five out of six guidelines, referenced management across all three categories of care as outlined in Figure 2 [32, 35, 42, 43, 45, 46, 47, 49]. The remaining 11 articles demonstrated gaps in addressing one or more management principles. The most frequent management area omitted was that of holistic care, with the primary focus of a majority of articles being pharmacological agents and referral of patients to other services.

### 3.3 Critical Appraisal of Individual Sources of Evidence

**FIGURE 3:**
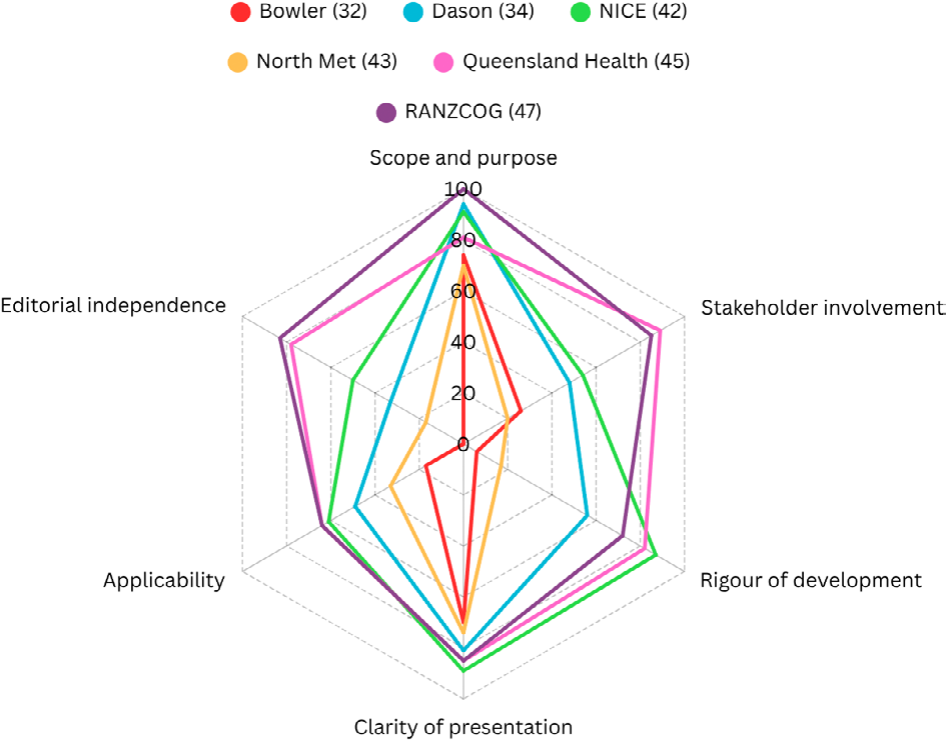
Radar chart of AGREE II domains.

Using the AGREE II instrument, we assessed guidelines across six domains and provided an overall score, evaluating their individual and comparative quality. Using the relevant JBI appraisal checklist, the expert opinion, case reports and qualitative study were evaluated with a determination of the quality of each result provided. Detailed results of the AGREE II appraisal are available in Appendix S3; a summary of the six domain scores is shown in Figure 3. The detailed JBI critical appraisal results are provided in Appendix S4. The expert opinion, two case reports and the qualitative study were assessed as being of reasonable quality by both independent reviewers. Results from the AGREE II appraisal showed varying results, with the Mater Mothers Hospital guideline scoring the lowest across domains and the RANZCOG guideline scoring the highest. All guidelines displayed their information in a clear manner according to results from domain three (clarity of presentation). Domain one (scope and purpose) was shown to be of the highest quality, with all guidelines scoring at or above 70%. However, domain five (applicability), deemed the most relevant domain given the aim of the review, was shown to be of overall low‐medium quality, with scores ranging from 17% to 64%. The sixth domain of editorial independence showed to vary greatly, with a range of scores between 0% and 83%.

## 4 Discussion

This scoping review sought to investigate how clinicians in the acute, non‐specialist setting manage patients who present with gynaecological CPP. We identified 19 articles from five countries between 1989 and 2024. Our findings illustrate that the role within the multidisciplinary team of clinicians who manage gynaecological CPP is poorly defined for those who work in the emergency and out‐of‐hospital setting. The majority of the included literature focused on various pharmacological options for pain management. In contrast, nonpharmacological strategies were mentioned less frequently, and psychosocial considerations were even more rarely addressed.

The most common intervention recommended by the majority of articles was the administration of NSAIDs to relieve pain, despite limited evidence demonstrating their effectiveness [50]. One article recommended NSAIDs for endometriosis associated pain while also having a qualifier regarding their potentially limited effectiveness [40]. The mechanism of action of NSAIDs is to block prostaglandin (PGs) synthesis through the inhibition of two cyclooxygenase (COX) enzymes, effectively providing relief of inflammatory pain [51]. In gynaecological CPP, NSAIDs have been shown to be effective for many patients in dysmenorrhea related pain or generalised menstrual cramping [52]. However, approximately 18% of women with dysmenorrhea receive limited analgesic benefits from NSAIDs, and the efficacy of NSAIDs in other conditions associated with gynaecological CPP is even further limited [53]. Despite some studies suggesting pain associated with endometriosis is caused by prostaglandin synthesis, NSAIDs have been shown to be largely ineffective or severely limited in this cohort [50]. Positives of using NSAIDs for gynaecological CPP in the acute setting include their favourable short‐term side effect profile, high acceptability, availability and low cost [54]. However, gastrointestinal side effects such as nausea, vomiting, cramping and diarrhoea may exacerbate gynaecological CPP and increase with prolonged use [50].

Hesitance or complete avoidance of using opioids in the treatment of gynaecological CPP was expressed by a number of articles, some without additional clarification of what treatment would be more appropriate or effective. Majority of included publications arose from the United States of America which may explain the wide expression of opioid hesitance given the well‐documented opioid epidemic influencing provider prescriptions [55]. Although opioids are largely not recommended as a first line treatment in chronic non‐cancer pains such as conditions associated with gynaecological CPP, they can be utilised in a responsible manner as part of a multimodal, stepwise and time‐limited approach [56]. Whilst research regarding opioid use by paramedics for chronic pain is limited, opioids in conjunction with multimodal and non‐pharmacological therapies, have been shown to be an effective and safe method of analgesia in the out‐of‐hospital setting [57, 58]. However, some multimodal therapies as suggested by broader evidence and included literature render this approach impractical in the out‐of‐hospital setting due to various resource and medicolegal constraints [59, 60]. In Australia, paramedics carry limited analgesics indicated for pain management (typically simple oral analgesics: paracetamol and ibuprofen, Schedule 4 medications: methoxyflurane and Schedule 8 opioids: morphine and fentanyl) [61]. Paramedics either do not carry or are not indicated to administer medications across other pharmacological classes (antidepressants, anxiolytics, muscle relaxants) recommended in the management of gynaecological CPP which may constrain them to using opioids [4]. Additionally, paramedics must operate within legislated scope of practice and jurisdictional guidelines under oversight from the Australian Health Practitioner Regulation Agency (AHPRA) [59, 60].

Majority of literature included in this review referenced pharmacological agents with less attention directed toward the importance of non‐pharmacological and holistic approaches. Though pharmacological analgesic agents are often indicated for treating pain in acute settings, the broader literature strongly emphasises implementation of non‐pharmacological techniques and person‐centred care (PCC) enacted through a biopsychosocial lens in the management of chronic pain [62, 63, 64]. Many commonplace non‐pharmacological therapies reserved for acute and traumatic injuries in the acute setting, such as ice, elevation and splinting, may not be applicable to those presenting with exacerbations of gynaecological CPP [64]. However, other recommended psychological techniques for acute pain management, such as distraction, imagery, deep breathing and relaxation, may be of value to this patient cohort [64]. Additionally, local active warming in women with pelvic pain in the out‐of‐hospital setting has been shown to alleviate pain in some cases [65].

Through application of PCC, clinicians can view the ‘whole person’ and consider how pain affects daily functioning, as well as biological, psychological and social wellbeing, enabling tailored interventions aligned with an individual's priorities, values and life context [62, 63]. In the acute care setting, clinicians can implement PCC principles within their management of gynaecological CPP through patient advocacy, a collaborative communication style and culturally appropriate practices [62, 66]. Emphasising delivery of empathetic and affirmative communication whilst implementing reassurance techniques has also been shown to improve patient outcomes in those with chronic and/or nociplastic pain and is translatable to providers in this setting [67, 68].

Despite effectiveness, more complex physical interventions such as physical therapy, acupuncture, TENS and hypnosis are less practically applied to acute care settings due to time, resource and training constraints [64, 65, 67, 68, 69]. However, to enhance the provision of holistic care, where possible, clinicians in the acute care setting should consider these therapies adjunctively through referral or consult.

An essential component of effective pain management that was not sufficiently covered in the included literature is the importance of a thorough pain assessment. Despite their widespread use, unidimensional tools such as the numerical rating scale when used without adjuncts may be insufficient in the assessment and subsequent management of chronic pain including conditions associated with gynaecological CPP [70, 71]. Evaluating pain through a biopsychosocial lens with tools which consider all dimensions of health may assist clinicians in selecting appropriate interventions, guide future care and referral and reduce stigma perpetuated by healthcare providers [70, 72]. Further and enhanced training in this area such as online training modules and workshops could provide HCP's with the necessary tools to better address patients biopsychosocial needs in the emergency and out‐of‐hospital setting [73].

### 4.1 Implications for Practice and Future Research Considerations

Given the various barriers patients face in accessing specialised care, clinicians in both the emergency and paramedic practice setting need to be educated and prepared to manage patients with gynaecological CPP as part of the multidisciplinary team. HCP's in these settings may serve as the first point of contact for many patients or be utilised when patients are experiencing an acute crisis, exemplifying their crucial role in patient care. A recent review of the scope of practice of Australian primary care HCP's strongly advocated for enhancing the multidisciplinary team approach [74].

Recent Australian federal and state‐led strategies focused on women's health and gynaecological CPP, such as the 2018 ‘National Action Plan for Endometriosis’ (NAPE), which includes the opening of 22 specialised endometriosis clinics across Australia, and the Victorian State ‘Women's Health and Wellbeing Program,’ which will involve a state inquiry into women's pain, present opportunities to explore and develop pathways for clinicians in the acute, non‐specialist setting [75, 76].

As demonstrated by the scant mention in this review, more attention must be directed toward practitioners in the paramedic practice setting, given their unique and important position in patient care. Initiatives such as the NAPE, aim to improve awareness and understanding of endometriosis among ‘health professionals working at every stage in the clinical pathway’ inclusive of emergency department staff as outlined in criteria 4 [75]. However, no reference to paramedics or paramedicine is made in the action plan, indicating a fundamental lack of understanding in how healthcare may be sought by those in acute need. In addition to the management of the symptom of pain, clinicians in the acute care setting must consider that pelvic pain can be symptomatic of underlying serious or emergency pathologies including ectopic pregnancy and ovarian cyst rupture or torsion. Whilst not a focus of this study, these differential diagnoses must be investigated as they may mimic or coexist with CPP. Patients meeting ‘red‐flag’ criteria, such as those outlined by the Royal College of Obstetricians and Gynaecologists, must promptly recognised and escalated by clinicians in the acute care setting among the multidisciplinary team of HCP's managing this cohort of patients [77].

The scope of practice of paramedics in Australia is rapidly expanding; milestones including tertiary education standards, the 2018 AHPRA recognition of paramedics and emerging Community Paramedic/Paramedic Practitioner models (which focus on the delivery of holistic care) have altered the landscape in which paramedics operate and provide care [60, 78]. In conjunction with increased reliance on acute care services for chronic health complaints, providers in this setting have the potential to invaluably contribute to better, holistic CPP management [60]. There is great opportunity for future research to be enriched by the unique perspective of the acute care setting within biomedical, psychological and social domains. Research gaps and future opportunities within the acute care setting related to each health domain are outlined in Table 2.

**TABLE 2:** Research gaps and future opportunities.

Domain	Identified acute care setting gap	Research opportunity
Biomedical	Limited evidence regarding thorough pain assessments for chronic pain. Restricted availability and indication for utilising multimodal approach with various medication classes. Time/resource constraints limiting application of non‐pharmacological options.	Can providers in the acute care setting deliver comprehensive chronic pain assessments like the Brief Pain Inventory within time/resource constraints for CPP? How can paramedics utilise tools readily available to them in the management of CPP (access to heat therapy/positioning/stretching). Are NSAIDs other than ibuprofen (mainstay in acute care settings) appropriate or applicable to be administered by acute care clinicians?
Psychological	Scant mention of psychological interventions including mental health first aid for CPP‐related distress. Poor integration of understanding psychological predispositions, elements and impacts for CPP.	How effective are clinicians at identifying psychological distress and associated factors in patients with CPP? Can acute care providers deliver brief psychological techniques to patients with CPP‐related distress (validation, relaxation, deep breathing)
Social	Referrals centred around gynaecologists and pain specialists, not other potentially relevant allied health professionals (social workers/occupational therapists/psychologists)	How can paramedic‐initiated referrals be optimised? What insights from seeing patients in their home environment (maladaptive coping strategies, social support, access to specialised care) can inform holistic CPP management?

## 5 Limitations

Only English‐language articles were eligible for inclusion in this review due to resource constraints. This potentially skews the findings to a Western perspective, reinforcing existing disparities within research on gynaecological CPP [79].

Much of the included literature was categorised as independent, ‘clinical review’ articles of which authors were unable to identify a suitable appraisal tool. Nevertheless, this underscores the prevalence of non‐evidence‐based or non‐peer‐reviewed recommendations being made within the management of gynaecological CPP in the emergency setting.

## 6 Conclusions

This scoping review underscores the urgent need to define and implement clear roles for clinicians managing gynaecological CPP exacerbations in acute, non‐specialist settings. It reveals substantial gaps in the literature related to best‐practice pain assessment, pharmacological and non‐pharmacological management and clinician education in this area alongside variable and often poor‐quality guideline recommendations. Effective pain management in CPP is dynamic, relying on an approach that incorporates healthcare providers of multiple disciplines and across all dimensions of health. Although there are inherent limitations within acute, non‐specialist settings, there are opportunities for healthcare providers to expand their knowledge and scope of practice. By shifting from outdated, unidimensional pain management strategies, clinicians can improve pain assessment and relief, enhancing patient outcomes.

## Funding

The authors have nothing to report.

## Conflicts of Interest

The authors declare no conflicts of interest.

## Supporting information


**Appendix S1:** PRISMA‐ScR checklist.
**Appendix S2:** Database and grey literature search terms.
**Appendix S3:** AGREE II appraisal results for included guidelines.
**Appendix S4:** JBI critical appraisal results for case studies, expert opinion and qualitative study.

## Data Availability

The data that support the findings of this study are openly available in Open Science Framework Search Strategy at https://osf.io/ztdws/?view_only=dabde2786df643ef914a8ca5018d02cf.
